# Synbiotic modulation of adult gut microbiome by 2′-fucosyllactose and *Bifidobacterium longum* subsp. *infantis* EFEL8008

**DOI:** 10.20517/mrr.2025.35

**Published:** 2025-07-22

**Authors:** Dong Hyeon Lee, Hyunbin Seong, Seul-Ah Kim, Nam Soo Han

**Affiliations:** Director of Brain Korea 21 Center for Smart GreenBio Convergence and Sustainable Regional Development, Division of Animal, Horticultural, Food Sciences, Chungbuk National University, Cheongju 28644, Republic of Korea.; ^#^Authors contributed equally.

**Keywords:** 2′-Fucosyllactose, *Bifidobacterium longum* subsp.* infantis*, synbiotics, *in vitro* digestion, *in vitro* fecal fermentation

## Abstract

**Aim:** This study aimed to evaluate the combination of 2′-fucosyllactose (2′-FL) and *Bifidobacterium longum* subsp. *infantis* (*B. infantis*) EFEL8008 as a synbiotic pair for adult gut health, using an *in vitro* digestion and fecal fermentation model.

**Methods:** The resistance of 2′-FL to digestion was evaluated through simulated digestion encompassing oral, gastric, intestinal, and brush border membrane phases. Fecal fermentation was conducted using adult microbiota to investigate taxonomic and metabolic alterations following treatment with 2′-FL, EFEL8008, or their combination. Microbial composition was profiled using 16S rRNA gene sequencing and quantitative PCR targeting *B. infantis*. Short-chain fatty acids (SCFAs) and trimethylamine (TMA) levels were quantified by ^1^H-NMR.

**Results:** A total of 86.67% of 2′-FL remained intact after digestion, demonstrating its resistance to digestion throughout the upper gastrointestinal tract. The synbiotic combination significantly increased *Bifidobacterium* abundance and improved alpha diversity compared to single treatments. Heat tree and correlation analyses indicated selective enrichment of commensal taxa including *Bifidobacterium* and *Lactobacillus*, accompanied by a reduction in the abundance of potentially pathogenic genera such as *Escherichia-Shigella*. In addition, co-treatment markedly elevated the concentrations of acetate, propionate, lactate, and butyrate, and suppressed the microbial conversion of betaine to TMA, suggesting a favorable metabolic outcome.

**Conclusion:** These results demonstrate that the synbiotic combination of 2′-FL and EFEL8008 promotes beneficial microbial modulation, enhances metabolite production, and supports gut health, highlighting its potential as a next-generation synbiotic strategy.

## INTRODUCTION

2′-Fucosyllactose (2′-FL), one of the most predominant human milk oligosaccharides (HMOs), consists of lactose and fucose linked via an α-(1,2)-glycosidic bond^[1]^. It is approved as “generally recognized as safe” (GRAS) by the FDA and as a novel food by the European Food Safety Authority (EFSA). 2′-FL exhibits several bioactive properties, including prebiotic, antibacterial, antiviral, and immune-supporting effects^[2,3]^. It plays a crucial role in infant health by selectively supporting beneficial bacteria in the gut microbiota and has been shown to reduce diarrhea in breastfeeding infants, underscoring its importance in early life nutrition^[4,5]^. Recently, due to great interest in commercial applications as most promising oligosaccharides, a technology for mass production by cloning FUT2 (α-1,2-fucosyltransferase gene), a core enzyme for 2′-FL biosynthesis, has been developed using *Corynebacterium glutamicum* and *Escherichia coli* fermentation^[6,7]^. Therefore, the development of probiotics that can effectively use 2′-FL as a substrate represents a promising avenue for creating health-promoting synbiotics.

*Bifidobacterium longum* subsp. *infantis* (*B. infantis*) is a Gram-positive, heterofermentative, anaerobic bacterium that plays a pivotal role in the infant gut microbiota, particularly during the first year of life^[8]^. It colonizes the infant gut shortly after birth and is uniquely capable of utilizing HMOs, specifically 2′-FL, as a primary carbon source^[9,10]^. Unlike other bacteria that metabolize HMOs extracellularly, *B. infantis* internalizes them prior to degradation, reducing opportunities for cross-feeding and limiting the growth of competing microorganisms^[9,11]^. This distinct metabolic capability contributes to the health benefits of *B. infantis*, including immune modulation, enhanced gut barrier function, reduced gastrointestinal inflammation, and the production of beneficial metabolites such as acetate^[12-14]^.

A synbiotic is defined as “a mixture comprising live microorganisms and substrate(s) selectively utilized by host microorganisms that confers a health benefit on the host”^[15]^. In detail, synbiotics can beneficially affect the host by improving the survival and implantation of live microbial dietary supplements in the gastrointestinal tract^[16]^. Over the past decade, synbiotics have been reported to more effectively reshape the gut microbiota and confer greater health benefits compared to probiotics or prebiotics alone^[17-19]^. In particular, they have been associated with improvements in inflammatory responses, liver enzyme markers, insulin resistance, and lipid metabolism through modulation of functional gut microbial populations^[18,19]^. For instance, yogurt with *Lacticaseibacillus casei* or *Lactobacillus helveticus* combined with prebiotics such as polydextrose or lactitol has been shown to positively impact digestive health by modulating gut microbiota composition^[20]^. In addition, cheeses containing *Lacticaseibacillus paracasei* or *Bifidobacterium lactis* paired with fructooligosaccharides (FOS) have demonstrated benefits for digestive health^[21]^. Thus, developing a suitable synbiotic supplement requires careful selection to achieve either synergistic or complementary effects between probiotics and prebiotics^[22]^. Given this background, a synbiotic combining *B. infantis* and 2′-FL has strong potential to modulate the gut environment. Recent advances in microbiome research have clarified the mechanistic pathways by which probiotics and prebiotics influence host health. Probiotics modulate microbial composition and function, which in turn affects intestinal homeostasis through multiple axes, including epithelial barrier reinforcement, short-chain fatty acid (SCFA) production, and suppression of pathobionts. For example, *Bifidobacterium* and *Lactobacillus* species have been shown to upregulate tight junction proteins (e.g., occludin and ZO-1), thereby enhancing gut barrier integrity and reducing microbial translocation - key factors implicated in inflammatory bowel disease (IBD) progression^[13,14]^. In parallel, prebiotics such as 2′-FL promote the growth of saccharolytic bacteria that generate SCFAs, which activate host G-protein coupled receptors (GPCRs) such as FFAR2 and FFAR3. These pathways influence host lipid metabolism, glucose homeostasis, and immune modulation, providing therapeutic implications for metabolic syndrome and chronic low-grade inflammation^[23,24]^. Moreover, recent studies highlight that synbiotics can inhibit microbial genes involved in trimethylamine (TMA) formation, such as *cutC* and *cntA*, reducing downstream production of pro-atherogenic trimethylamine-N-oxide (TMAO)^[25,26]^. These mechanisms collectively underscore the potential of rational synbiotic combinations to achieve targeted modulation of the gut ecosystem and mitigate disease-related microbial pathways.

Therefore, this study aimed to evaluate the synbiotic effects of 2′-FL and *B. infantis* EFEL8008, using an *in vitro* human digestion and fermentation model. The *in vitro* model system offers several advantages for synbiotic research, including controlled environments that enable detailed analysis of gut-specific interactions and microbial activity. The EFEL8008 strain used in this study was originally isolated from the feces of a breastfed infant and was selected based on its robust growth on 2′-FL as a sole carbon source compared to multiple strains of *Bifidobacterium*, including *B. infantis*. To further enhance this capability, the strain was subjected to adaptive laboratory evolution (ALE), resulting in improved 2′-FL utilization^[27]^. To assess microbial shifts, fecal fermentation experiments were conducted under anaerobic conditions, and *B. infantis* was selectively quantified via RT-qPCR. *In vitro* fecal fermentation was subsequently conducted to analyze microbial composition, and the abundance of *B. infantis* was quantified by RT-qPCR. Additionally, ^1^H-NMR analysis was conducted to profile metabolites generated during fermentation. This study is the first to propose a synbiotic pairing of *B. infantis* and 2′-FL that enables stable colonization and metabolic activity in adult fecal microbiota, offering a novel strategy for next-generation synbiotic design.

## METHODS

### Materials and bacterial strains

The 2′-FL (purity ≥ 99%) used in this study was generously supplied by Advanced Protein Technologies Corp. (Suwon, Korea). *B. infantis* EFEL8008, originally isolated from infant feces, was acquired from the Korean Collection for Type Cultures (KCTC; accession number KCTC15117BP). Vitamin K_1_ was sourced from Wako Chemicals (Osaka, Japan). L-cysteine hydrochloride, magnesium sulfate heptahydrate (MgSO_4_·7H_2_O), bile salts, hemin, resazurin, α-amylase (from human saliva, Type IX-A), pepsin (from porcine mucosa, product code P7000), pancreatin (from porcine pancreas, product code P7545), and formic acid were obtained from Sigma-Aldrich/Merck (Darmstadt, Germany). Peptone water and yeast extract were purchased from BD Biosciences (Franklin Lakes, NJ, USA). The following inorganic salts used in the experiments - CaCl_2_·2H_2_O, K_2_HPO_4_, KH_2_PO_4_, and NaCl - were sourced from Junsei Chemical Co. (Tokyo, Japan). In addition, Tween 80 was supplied by VWR (Radnor, PA, USA). All other chemicals employed in this study were of analytical reagent grade unless stated otherwise.

### Growth characteristics of *B. infantis* EFEL8008 with 2′-FL

To assess the growth potential of *B. infantis* EFEL8008 on 2′-FL as the sole carbohydrate source, the strain was cultured in 10 mL of glucose-free MRS medium supplemented with 0.05% (w/v) L-cysteine and 1% (w/v, equivalent to 20.47 mM) 2′-FL. Cultivation was carried out for 24 h at 37 °C under anaerobic conditions in a chamber system (Coy Laboratory Products, Grass Lake, MI, USA). Bacterial growth was monitored by measuring optical density at 600 nm using a Synergy HTX spectrophotometer (BioTek Instruments, Winooski, VT, USA), and viable counts were determined via standard plate counting. Residual 2′-FL concentrations were analyzed by high-performance liquid chromatography (HPLC) on a YOUNG-LIN M720 system (Seoul, Korea), equipped with a refractive index detector and an Aminex HPX-87H column (Bio-Rad, Hercules, CA, USA). The column was maintained at 37 °C, with 5 μL of each sample injected. The mobile phase consisted of 5 mM sulfuric acid in deionized water, delivered at a flow rate of 0.6 mL/min. All solvents used in the analysis were of HPLC-grade quality.

### *In vitro* digestibility of 2′-FL

The *in vitro* digestion procedure for 2′-FL was adapted with modifications from a previously established protocol^[28]^. For the oral digestion step, simulated salivary fluid (SSF) and α-amylase (1,500 U/mL; Type IX-A, human saliva origin, Sigma) were added to the substrate, and the reaction was maintained at 37 °C for 2 min to mimic physiological conditions. Gastric digestion was simulated by adding simulated gastric fluid (SGF, pH 3.0) and pepsin (25,000 U/mL; derived from porcine gastric mucosa, Sigma) to the oral-phase product, followed by incubation at 37 °C for 2 h. For the intestinal phase, 4 mL of the gastric digest was mixed with simulated intestinal fluid (SIF) and pancreatin (100 U/mL; porcine source, Sigma), and the mixture was held at 37 °C for an additional 2 h. The final digestion step involved treatment with brush border membrane vesicles (BBMV), which were obtained by carefully excising the ileal mucosa of porcine small intestine and homogenizing the tissue in buffer using a PTFE pestle-glass tissue grinder (φ13 × L93 mm, Wheaton, NJ, USA)^[29]^. Magnesium chloride (MgCl_2_) was incorporated into the homogenized mucosal suspension to reach a final concentration of 10 mM, after which the mixture was allowed to stand for 15 min. The suspension was subsequently centrifuged, and the pellet obtained was resuspended in phosphate-buffered saline (PBS) and further homogenized to prepare the BBMV fraction. Freshly prepared SIF was then added to the small intestinal digest along with the BBMV enzyme at a final maltase activity of 2.89 U/mL. The reaction mixture was incubated at 37 °C for 4 h.

### *In vitro* fecal fermentation

To investigate the impact of EFEL8008 and 2′-FL on gut microbial composition, anaerobic batch fermentation was conducted using a modified protocol adapted from a previously established method^[30]^. Nineteen adult volunteers who had not consumed antibiotics, probiotics, or prebiotics in the preceding three months and had no recent gastrointestinal disorders provided fresh stool samples. These samples were immediately processed upon arrival and maintained at 4 °C in sealed containers to minimize compositional shifts. All procedures involving human materials were reviewed and approved by the Institutional Review Board of Chungbuk National University (approval number: CBNU-202211-BR-0238). To prepare the inoculum, fecal matter was homogenized with PBS at a 1:10 ratio. A fermentation medium was formulated to support microbial growth, composed of peptone water (2 g/L), yeast extract (1 g/L), NaCl (0.1 g/L), phosphate salts, mineral components, bile salts (0.5 g/L), L-cysteine (0.5 g/L), hemin (50 mg/L), vitamin K1 (10 μL/L), Tween 80 (2 mL/L), and 0.05% resazurin as a redox indicator. The pH was standardized to 7.0, and the medium was pre-reduced under anaerobic conditions overnight. A total of 150 mL of the medium was inoculated with 1% (w/v) fecal slurry, and fermentation was initiated at 37 °C under constant stirring. EFEL8008 was introduced at a final concentration of 3.51 × 10^6^ CFU/mL in the EFEL8008 and EFEL8008 + 2′-FL groups, while 2′-FL was added at 1% (w/v) in the 2′-FL and EFEL8008 + 2′-FL groups. Samples (10 mL) were harvested at 12 and 24 h for microbial DNA extraction and metabolite profiling.

### 16S rRNA amplicon-based metagenome analysis and bioinformatics analysis

Microbial composition in the fecal fermentation samples was profiled based on 16S rRNA gene amplicon sequencing using the Illumina MiSeq platform (Illumina, San Diego, CA, USA). Amplification targeted the V3-V4 regions of the 16S rRNA gene using the primer pair 341-F (5′-CCTACGGGNGGCWGCAG-3′) and 785-R (5′-GACTACHVGGGTATCTAATCC-3′)^[31]^, in conjunction with Nextera indexing adapters. Raw sequence reads were subjected to quality trimming using Cutadapt and processed through the Deblur pipeline implemented in QIIME 2^[32,33]^. Taxonomic classification was carried out using a Naive Bayes classifier trained on the SILVA 132 reference database with weighted assignment. All bioinformatic analyses were performed in a dedicated Conda environment configured for QIIME 2 to ensure workflow reproducibility. Alpha diversity indices were visualized using the ggplot2 package in R, while taxonomic comparisons across groups were conducted through the MicrobiomeAnalyst online interface (https://www.microbiomeanalyst.ca/)^[34]^. Operational taxonomic units (OTUs) obtained from 16S rRNA gene sequencing were used for functional prediction using PICRUSt2 (v2.6.2).

### Monitoring of microbial changes by real-time q-PCR

To design primers specific to *B. infantis*, genome-wide sequence data were examined to identify gene regions uniquely present in this subspecies. Genomic DNA was isolated from the fermentation samples using a commercial bacterial DNA extraction kit (Solgent, Daejeon, Korea) according to the supplier’s instructions, and stored at -20 °C until further use. Genes exhibiting high sequence homology to those of other bacterial taxa were eliminated based on iterative MegaBLAST searches. Among the screened targets, the gene encoding sialidase was ultimately selected for species-level discrimination. Primer pairs were constructed to yield amplicons shorter than 175 bp, consistent with recommended fragment lengths (~150 bp) for optimal performance in real-time qPCR assays^[35]^. Amplification and detection of purified bacterial DNA by real-time qPCR were performed with the Exicycler^TM^96 (Bioneer, Daejeon, Korea). Each amplification reaction was carried out in triplicate for each fermentation sample using a primer set specific to the sialidase gene in *B. infantis* (Bi178-F: 5′-TCCTGTTCTTCGTCAAATCCTATGAC-3′ and Bi178-R: 5′-ATCCATGATTCGCTGTTCGTGA-3′). The amplification program was as follows: 50 °C for 5 min, 95 °C for 10 min, followed by 40 cycles of 95 °C for 15 s, 60 °C for 20 s, and a final cooling sequence at 4 °C for 20 min. As shown in Supplementary Figure 1A, the primer sequences were commonly present in the gDNAs of nine strains of *B. infantis*. A linear correlation was observed between the Ct values and the number of genome copies for all standard curves, with R^2^ values of 0.9984 [Supplementary Figure 1B-E]. These correlation coefficients demonstrated the reliability of the qPCR assays across a range of 5 log units. The slope of the standard curve for the gDNA of *B. infantis* was -3.43, closely aligned with a PCR efficiency of approximately 100% (-3.30).

### Metabolic analysis by ^1^H-NMR

Metabolite profiling of fermentation samples was performed using proton nuclear magnetic resonance (^1^H-NMR) spectroscopy^[36]^. Following fermentation, each sample was centrifuged at 16,000 ×*g* for 10 min, and the resulting supernatant was mixed in a 1:1 ratio with deionized water containing 10% deuterium oxide and 1 mM sodium 2,2-dimethyl-4-silapentane-1-sulfonic acid (DSS), yielding a final DSS concentration of 0.5 mM. The pH of the prepared solution was adjusted to 6.00 ± 0.01 using 2 M HCl or NaOH. A volume of 700 µL was transferred into a 0.5 mm NMR tube, and spectral data were acquired using a Varian INOVA 500 MHz NMR spectrometer (Varian Inc., Palo Alto, CA, USA). Spectral identification and quantification of metabolites were conducted with the Chenomx NMR Suite (version 6.1; Chenomx Inc., Edmonton, Alberta, Canada), utilizing the Processor and Profiler modules.

### Statistics

All experimental procedures were conducted in triplicate, and the resulting data are presented as mean values accompanied by standard deviations. Statistical comparisons between two groups were evaluated using independent two-sample t-tests with a two-tailed distribution. Analyses were carried out using IBM SPSS Statistics software (version 22; IBM Corp., Armonk, NY, USA). Visualization of figures was performed with GraphPad Prism software (version 8.0; GraphPad Software, San Diego, CA, USA). For core microbiome profiling, customized Python scripts (version 3.9) were utilized, incorporating functions from the pandas, seaborn, and matplotlib libraries.

## RESULTS

### Utilization of 2′-FL as the sole carbon source

**Figure 1:**
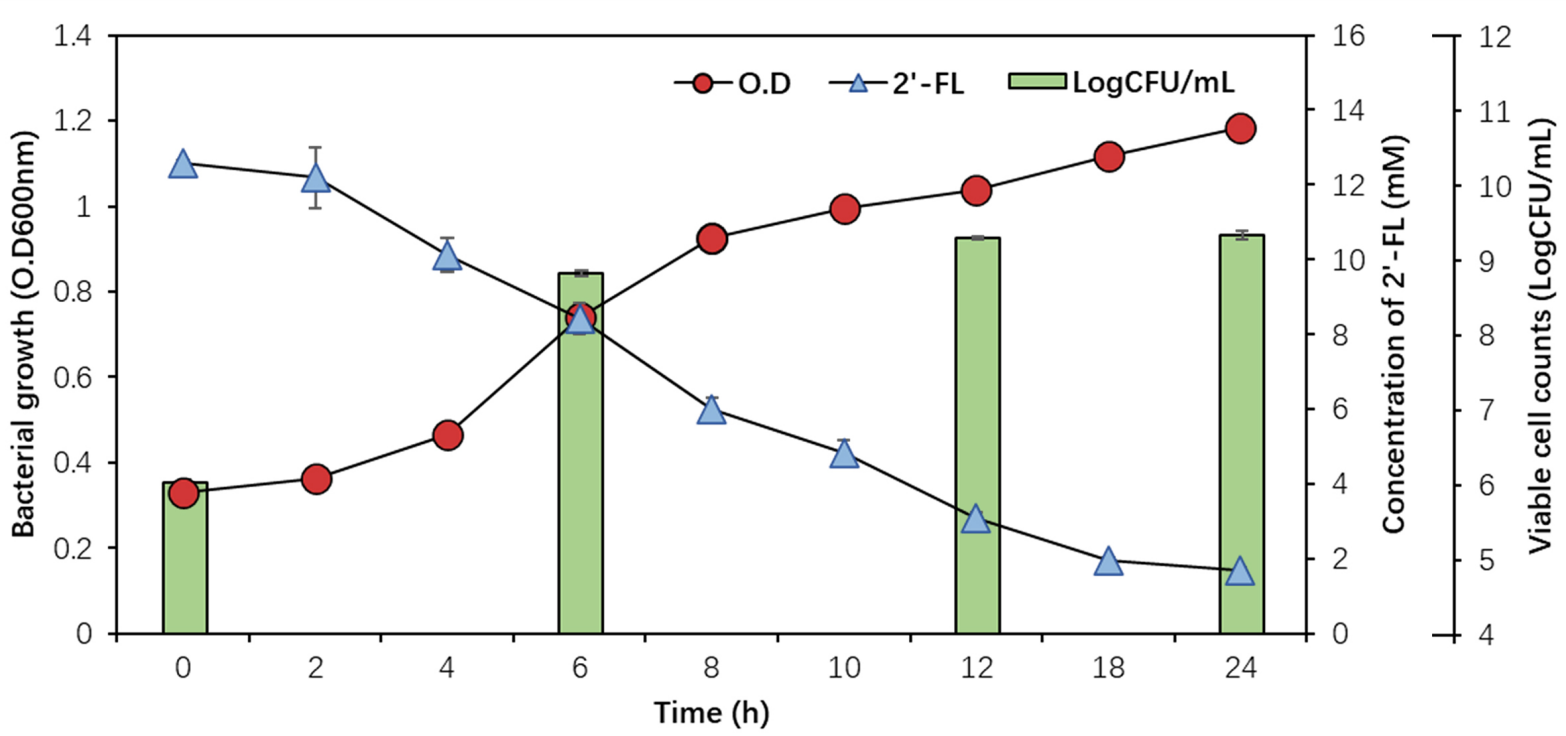
Cell growth of *B. infantis* EFEL8008 and residual concentrations of 2′-FL. The strain was cultured at 37 °C under anaerobic conditions in MRS medium supplemented with 1% (w/v, 12.59 mM) 2′-FL as the sole carbon source. Growth was monitored over 24 h by measuring OD600nm and viable cell count (LogCFU/mL), and 2′-FL concentrations were determined by HPLC. Error bars represent the standard deviation of triplicates. *B. infantis*: *Bifidobacterium longum* subsp. *infantis*; 2′-FL: 2′-fucosyllactose; HPLC: high-performance liquid chromatography.

To evaluate the ability of *B*. *infantis* EFEL8008 to utilize 2′-FL, cell growth and 2′-FL consumption were measured in MRS medium supplemented with 12.59 mM of 2′-FL as the sole carbon source. As shown in Figure 1, EFEL8008 exhibited a rapid growth, reaching a population level of OD600nm 1.18 ± 0.01 and 9.34 ± 0.05 Log CFU/mL after 24 h. Simultaneously, 2′-FL concentration was decreased (12.59 ± 0.08 to 1.70 ± 0.07 mM) in the culture medium after 24 h. This result revealed that EFEL8008 effectively metabolizes 2′-FL as a carbon source, supporting robust cell proliferation.

### *In vitro* digestion assay

The *in vitro* digestion model was utilized to investigate the hydrolysis resistance of 2′-FL by exposing it to oral fluid, SGF, SIF, and BBMV. As a result, 2′-FL was slightly hydrolyzed during the oral (0.80% ± 0.21%), gastric (-1.20% ± 0.63%), and intestinal (2.29% ± 1.24%) phases [Table 1]. Meanwhile, 2′-FL exhibited further enzymatic susceptibility in the BBMV phase, leading to additional degradation (11.65% ± 1.74%). These results imply that 86.67% of ingested 2′-FL could reach the large intestine intact after passing through the human gastrointestinal system.

**Table 1:** 2′-FL concentration before and after *in vitro* digestion

**Digestion phase**	**2′-FL concentration (mM)**	**Stepwise digestion ratio^a^ (%)**	**Overall digestion ratio (%)**
Before digestion	44.91 ± 0.20		13.33 ± 2.70
After oral phase	44.55 ± 0.12	0.80 ± 0.21	
After gastric phase	45.08 ± 0.25	-1.20 ± 0.63	
After small intestinal phase	44.05 ± 0.54	2.29 ± 1.24	
After BBMV phase	38.92 ± 1.04	11.65 ± 1.74	

Digestion of 2′-FL was assessed under *in vitro* artificial digestion conditions, simulating the mouth, stomach, and small intestine. ^a^Digestion ratio was calculated as 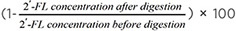. Values are mean ± standard deviation from triplicate determinations. 2′-FL: 2′-Fucosyllactose; BBMV: brush border membrane vesicles.

### Taxonomic analysis of microbial composition during *in vitro* fecal fermentation

**Figure 2:**
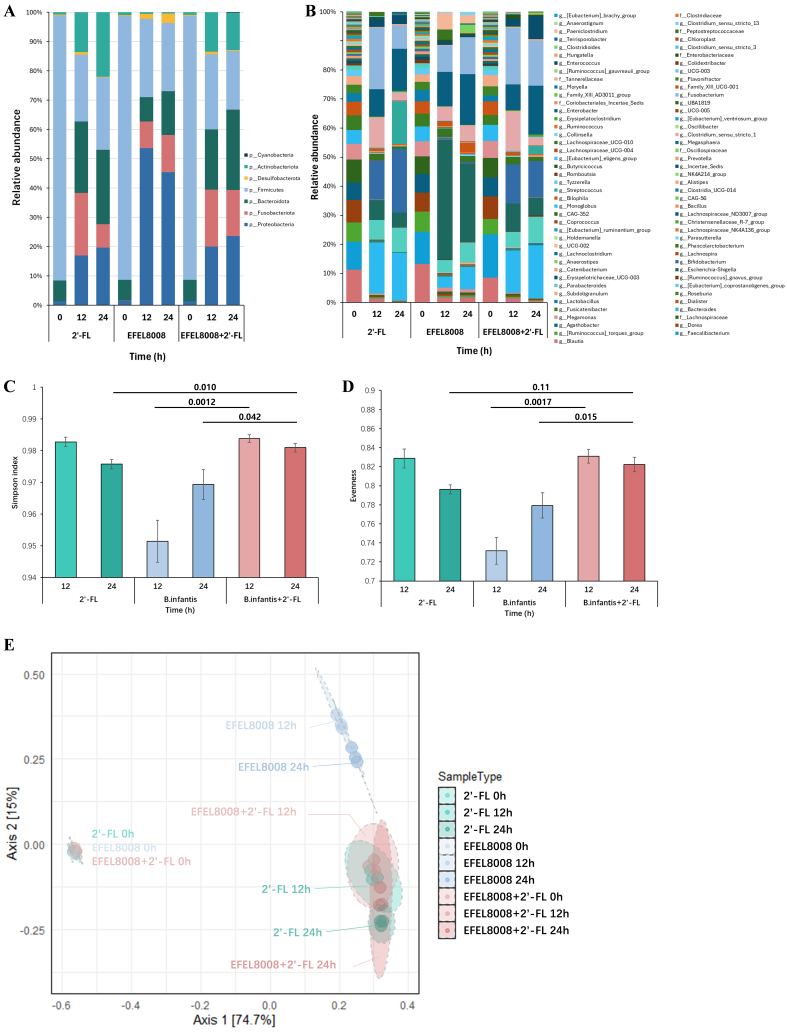
Microbial diversity analyses and microbial abundance profiling after *in vitro* fecal fermentation. Bacterial taxonomic composition at the (A) phylum and (B) genus levels; Alpha diversities of (C) Simpson Index and (D) evenness, with error bars representing the standard deviation from the mean (*n* = 3). Significant differences between groups are indicated by *P*-values; (E) PCoA plot of microbial communities based on Bray-Curtis distance in two dimensions. 2′-FL, addition of 1% (w/v) 2′-fucosyllactose; EFEL8008, addition of *B. infantis* EFEL8008 at 3.51 × 10^6^ CFU/mL; EFEL8008 + 2′-FL, addition of *B. infantis* EFEL8008 at 3.51 × 10^6^ CFU/mL and 1% 2′-FL. PCoA: Principal coordinate analysis; 2′-FL: 2′-fucosyllactose; *B. infantis*: *Bifidobacterium longum* subsp. *infantis.*

To evaluate the effects of 2′-FL and *B. infantis* EFEL8008 on the gut microbial composition, 16S rRNA gene-based taxonomic profiling was performed after *in vitro* fecal fermentation. At the phylum level, the relative abundance of Actinobacteria markedly increased in the 2′-FL (22.00% ± 1.16%) and EFEL8008 + 2′-FL (12.76% ± 3.05%) compared to the EFEL8008 (0.41% ± 0.02%) [Figure 2A]. At the genus level, *Bifidobacterium* was the most enriched taxon in both the 2′-FL (21.90% ± 1.15%) and EFEL8008 + 2′-FL (12.40% ± 3.06%) groups, while it remained minimal in EFEL8008 (0.39% ± 0.02%) [Figure 2B]. In terms of community structure, alpha diversity measured by the Simpson index and evenness was significantly higher in the 2′-FL and EFEL8008 + 2′-FL compared to EFEL8008 [Figure 2C and D]. Principal coordinate analysis (PCoA) based on Bray-Curtis dissimilarity further revealed distinct clustering of microbial communities, indicating that the co-administration of 2′-FL with EFEL8008 shifts both taxonomic abundance and overall diversity in a beneficial direction [Figure 2E].

### Modulation of microbial interactions by synbiotic pairing

**Figure 3:**
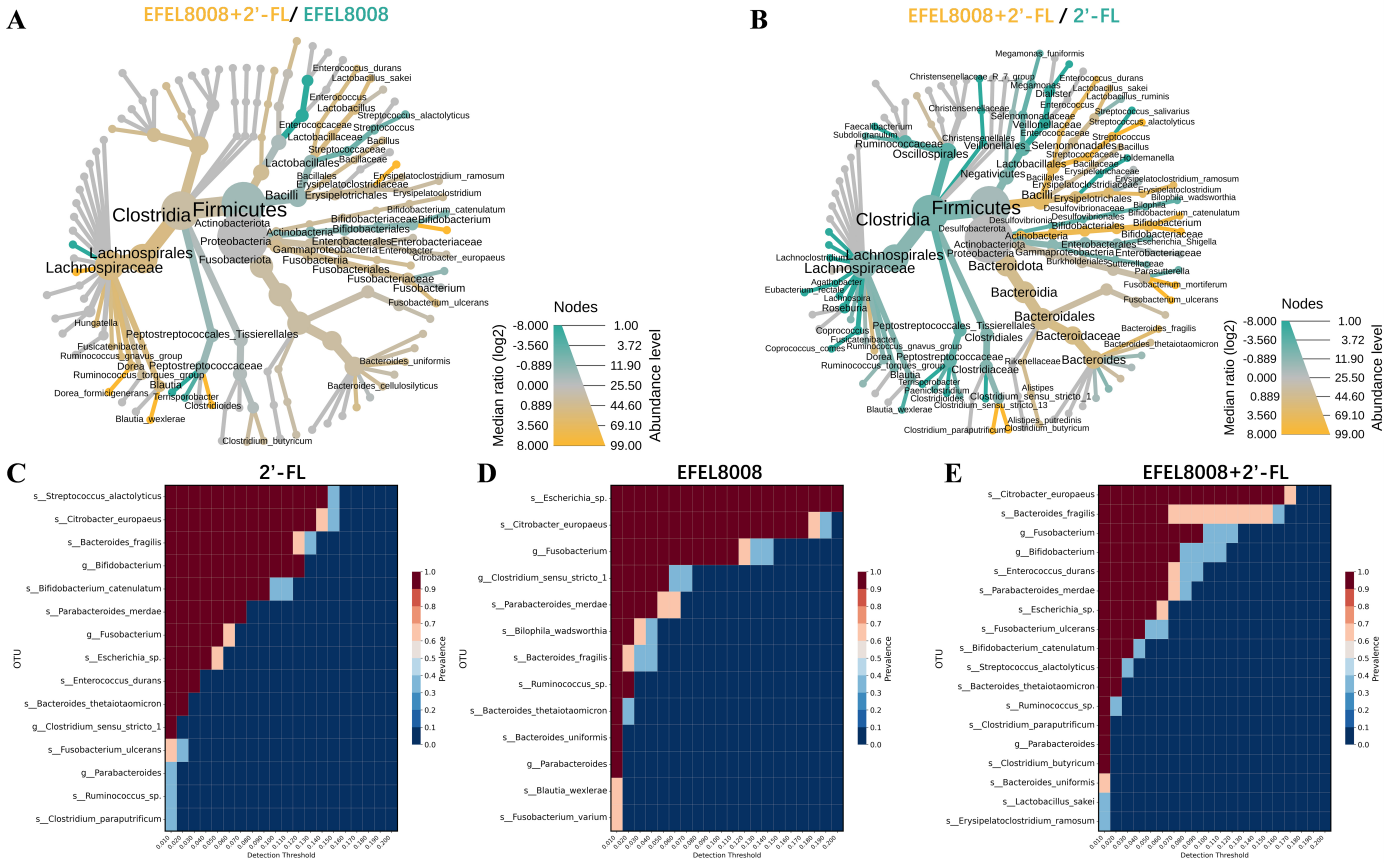
Comparison of microbial taxonomic compositions after *in vitro* fecal fermentation. Heat tree analysis compared microbial communities using median abundance and statistical differences based on the non-parametric Wilcoxon Rank Sum test. 2′-FL with *B. infantis* EFEL8008 was compared with either (A) *B. infantis* EFEL8008 or (B) 2′-FL at the species level after *in vitro* fermentation; (C-E) Core microbiome analysis showed species prevalence across detection thresholds for (C) 2′-FL, (D) *B. infantis* EFEL8008, and (E) 2′-FL with *B. infantis* EFEL8008 after *in vitro* fermentation. The color gradients indicated relative abundance and prevalence of the microbial species. 2′-FL, addition of 1% (w/v) 2′-fucosyllactose; EFEL8008, addition of *B. infantis* EFEL8008 at 3.51 × 10^6^ CFU/mL; EFEL8008 + 2′-FL, addition of *B. infantis* EFEL8008 at 3.51 × 10^6^ CFU/mL and 1% 2′-FL. 2′-FL: 2′-Fucosyllactose; *B. infantis*: *Bifidobacterium longum* subsp. *infantis.*

To investigate the microbial interaction patterns induced by the synbiotic combination, heat tree and core microbiome analyses were conducted. The heat tree visualization revealed that the EFEL8008 + 2′-FL exhibited a higher abundance of *Bifidobacterium* species compared to either EFEL8008 [Figure 3A] or 2′-FL [Figure 3B]. Notably, this group also showed a reduced abundance of potentially pathogenic taxa, such as *Escherichia-Shigella*, indicating a microbiota-stabilizing effect. Consistent with this, core microbiome analysis showed that *Bifidobacterium* was consistently enriched in the 2′-FL [Figure 3C], EFEL8008 [Figure 3D], and EFEL8008 + 2′-FL [Figure 3E] groups, with the highest abundance observed in the EFEL8008 + 2′-FL group. These observations suggest that synbiotic pairing facilitates selective engraftment of health-promoting microbes while limiting harmful species.

**Figure 4:**
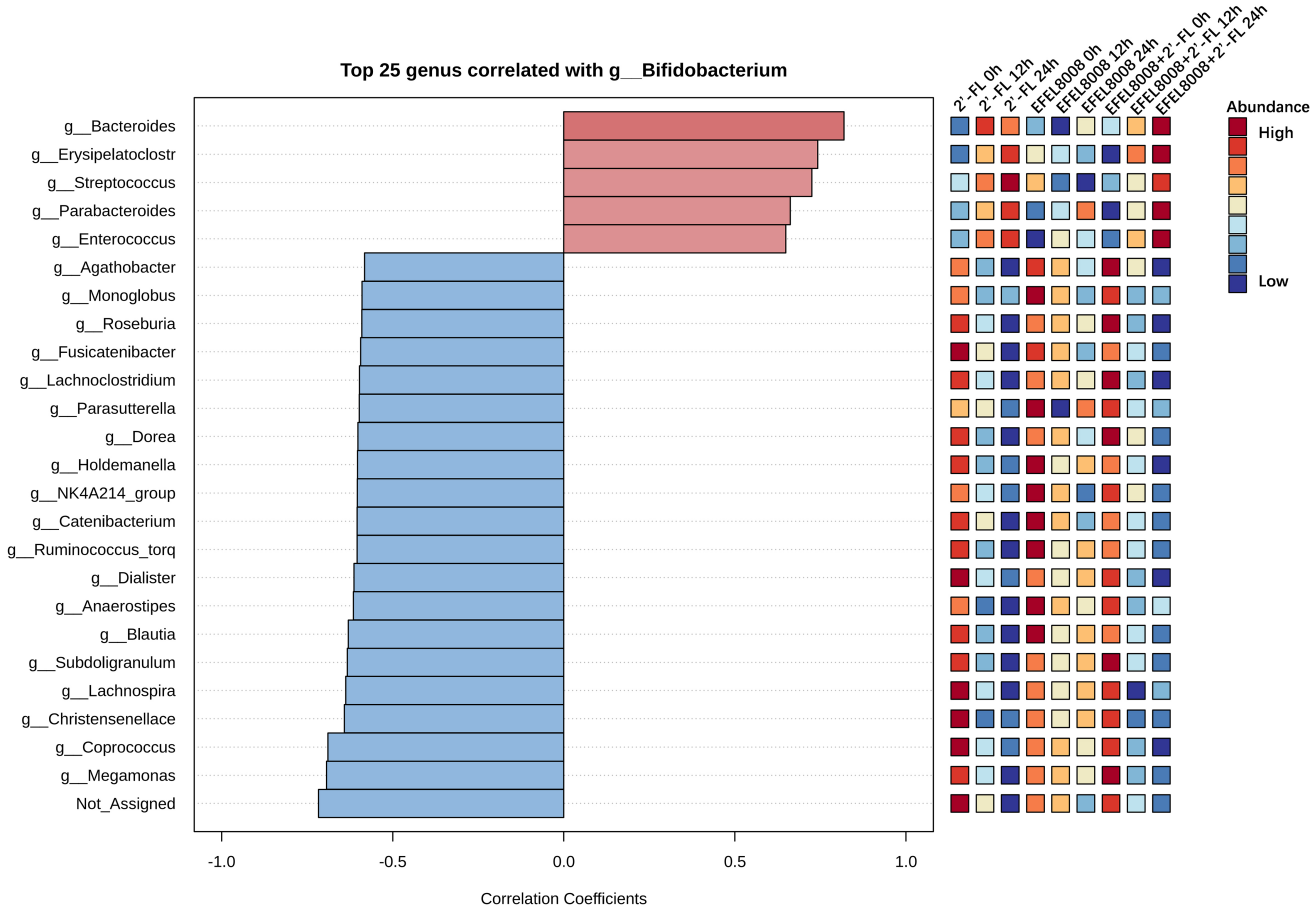
Pearson’s correlations using pattern search that identified the top 25 genera correlated with *Bifidobacterium*. Blue bars indicated negative correlations, while red bars indicated positive correlations, with deeper colors representing stronger correlations. The mini heatmap on the right displayed whether the abundance of each genus was higher (red) or lower (blue) in each group. 2′-FL, addition of 1% (w/v) 2′-fucosyllactose; EFEL8008, addition of *B. infantis* EFEL8008 at 3.51 × 10^6^ CFU/mL; EFEL8008 + 2′-FL, addition of *B. infantis* EFEL8008 at 3.51 × 10^6^ CFU/mL and 1% 2′-FL. 2′-FL: 2′-Fucosyllactose; *B. infantis*: *Bifidobacterium longum* subsp. *infantis.*

To further explore genus-level interactions, Pearson’s correlation analysis was performed using a pattern search approach centered on *Bifidobacterium*. As shown in Figure 4, *Bifidobacterium* abundance positively correlated with that of other beneficial genera such as *Lactobacillus* and *Bacteroides* in both the 2′-FL and EFEL8008 + 2′-FL. In contrast, strong negative correlations were observed with genera known to include pathogenic species, including *Escherichia* and *Clostridium*. These findings indicate that the combination of EFEL8008 and 2′-FL may contribute to a balanced microbial network by supporting commensal populations and reducing the abundance of opportunistic pathogens, with 2′-FL playing a predominant role in this effect.

### Monitoring *B. infantis* during the *in vitro* fermentation

**Figure 5:**
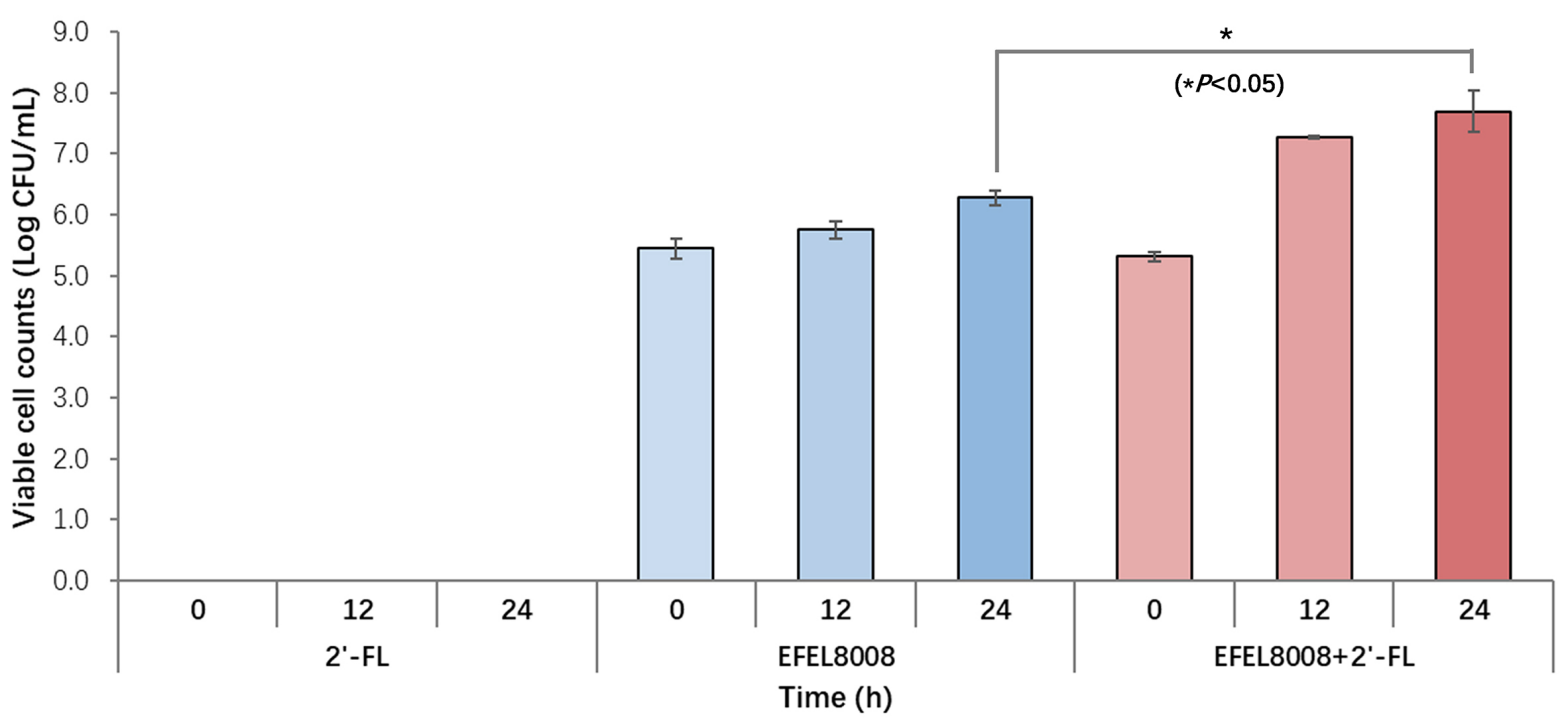
Quantification of *B. infantis* during *in vitro* fecal fermentation. *B. infantis* was quantified at 0, 12, and 24 h during *in vitro* fecal fermentation using real-time qPCR targeting the sialidase gene. Values represent the mean ± standard deviation (*n* = 3). *P* < 0.05 compared to EFEL8008. *B. infantis*: *Bifidobacterium longum* subsp. *infantis*; qPCR: quantitative polymerase chain reaction.

To specifically monitor *B. infantis* during fecal fermentation, we applied RT-qPCR methods as outlined in the Methods section. This approach enabled precise detection and targeted monitoring of *B. infantis* throughout the fermentation process. As shown in Figure 5, the EFEL8008 and EFEL8008 + 2′-FL, initially containing EFEL8008 at 3.51 × 10^6^ CFU/mL, stimulated the growth of *B. infantis* to 6.28 Log CFU/mL and 7.69 Log CFU/mL, respectively, after 24 h of fermentation. In addition, both EFEL8008 and EFEL8008 + 2′-FL showed a significant difference in the growth of *B. infantis* at 24 h (*P* = 0.011). However, no growth of *B. infantis* was observed in the 2′-FL group after 12 and 24 h of fermentation. These findings demonstrate that EFEL8008 achieves detectable engraftment and proliferation within adult fecal microbiota under synbiotic conditions, indicating broader ecological compatibility beyond the infant gut environment.

### Metabolite changes during the *in vitro* fecal fermentation

**Figure 6:**
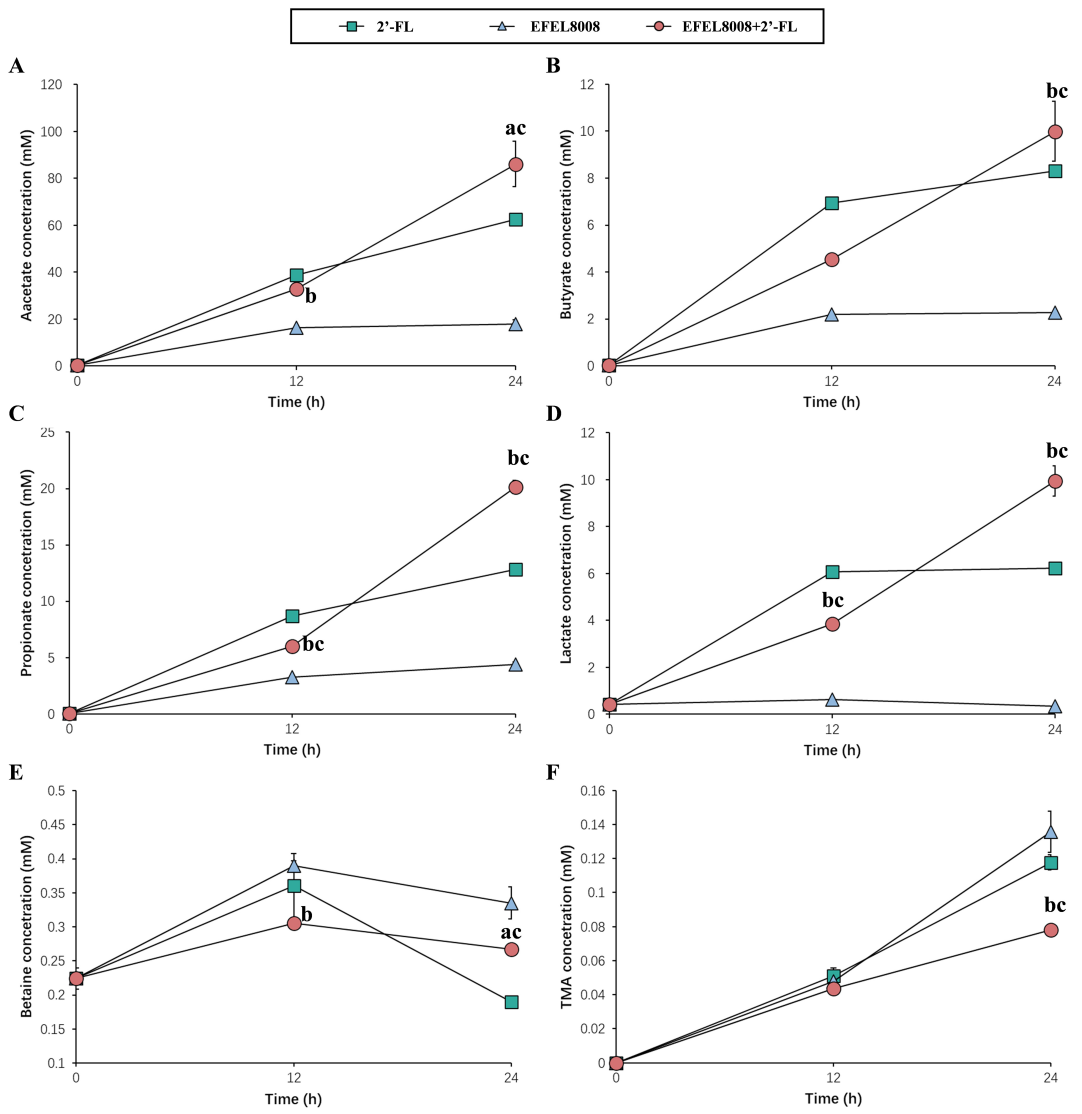
Changes in metabolite concentrations during *in vitro* fecal fermentation. (A) acetate, (B) butyrate, (C) propionate, (D) lactate, (E) betaine, and (F) TMA were analyzed. 2′-FL, addition of 1% (w/v) 2′-fucosyllactose; EFEL8008, addition of *B. infantis* EFEL8008 at 3.51 × 10^6^ CFU/mL; EFEL8008 + 2′-FL, addition of *B. infantis* EFEL8008 at 3.51 × 10^6^ CFU/mL and 1% 2′-FL. Error bars represent the standard deviation of triplicates. Significant differences are presented with EFEL8008 (a, *P* < 0.05; b, *P* < 0.01) or 2′-FL (c, *P* < 0.01). 2′-FL: 2′-Fucosyllactose; *B. infantis*: *Bifidobacterium longum* subsp. *infantis.*

^1^H-NMR analysis was conducted to evaluate metabolites, including SCFAs and TMA, produced during *in vitro* fecal fermentation of EFEL8008 and 2′-FL. As shown in Figure 6A-D, the EFEL8008 + 2′-FL significantly increased the production of acetate (86.12 ± 9.68 mM), propionate (20.14 ± 0.58 mM), lactate (9.94 ± 0.64 mM), and butyrate (10.00 ± 1.26 mM) after 24 h of fermentation. These values were notably higher compared to those observed in the EFEL8008 (33.10 ± 1.72, 7.42 ± 0.23, 2.28 ± 0.01, and 4.20 ± 0.04 mM, respectively) and 2′-FL (62.43 ± 1.32, 12.84 ± 0.40, 6.24 ± 0.09, and 8.31 ± 0.20 mM, respectively). Figure 6E and F shows a decrease in betaine concentrations across all samples as fermentation progressed, with a simultaneous increase in TMA. However, EFEL8008 + 2′-FL led to a lower conversion of betaine to TMA (0.08 ± 0.00 mM) compared to EFEL8008 (0.14 ± 0.00 mM) or 2′-FL (0.12 ± 0.01 mM) after 24 h. The increase in SCFAs and reduction in TMA observed under the combined treatment may be explained by taxon-specific microbial interactions. EFEL8008 is known to metabolize 2′-FL, producing metabolites such as acetate and lactate that can serve as substrates for cross-feeding. These intermediates may have promoted the growth of butyrate-producing taxa including *Anaerostipes* and *Faecalibacterium*, as reflected by the elevated butyrate levels [Figure 6D] and positive correlations with these genera [Figure 4]. Concurrently, the depletion of genera such as *Escherichia* and *Clostridium*, which are associated with microbial genes responsible for TMA formation (*cutC*, *cntA*), likely contributed to the reduction in TMA levels [Figure 6F]. While these interpretations remain hypothetical, they offer a plausible explanation based on the integrated metagenomic and metabolomic datasets. In summary, these results demonstrated that 2′-FL served as a fermentable substrate for gut microbiota, including *B. infantis* EFEL8008. The observed increase in SCFA production and reduction in microbial conversion of betaine to TMA were primarily driven by 2′-FL utilization, while the presence of EFEL8008 may have further supported these effects through its probiotic activity under synbiotic conditions.

### Functional gene prediction of microbial metabolism using PICRUSt2

**Figure 7:**
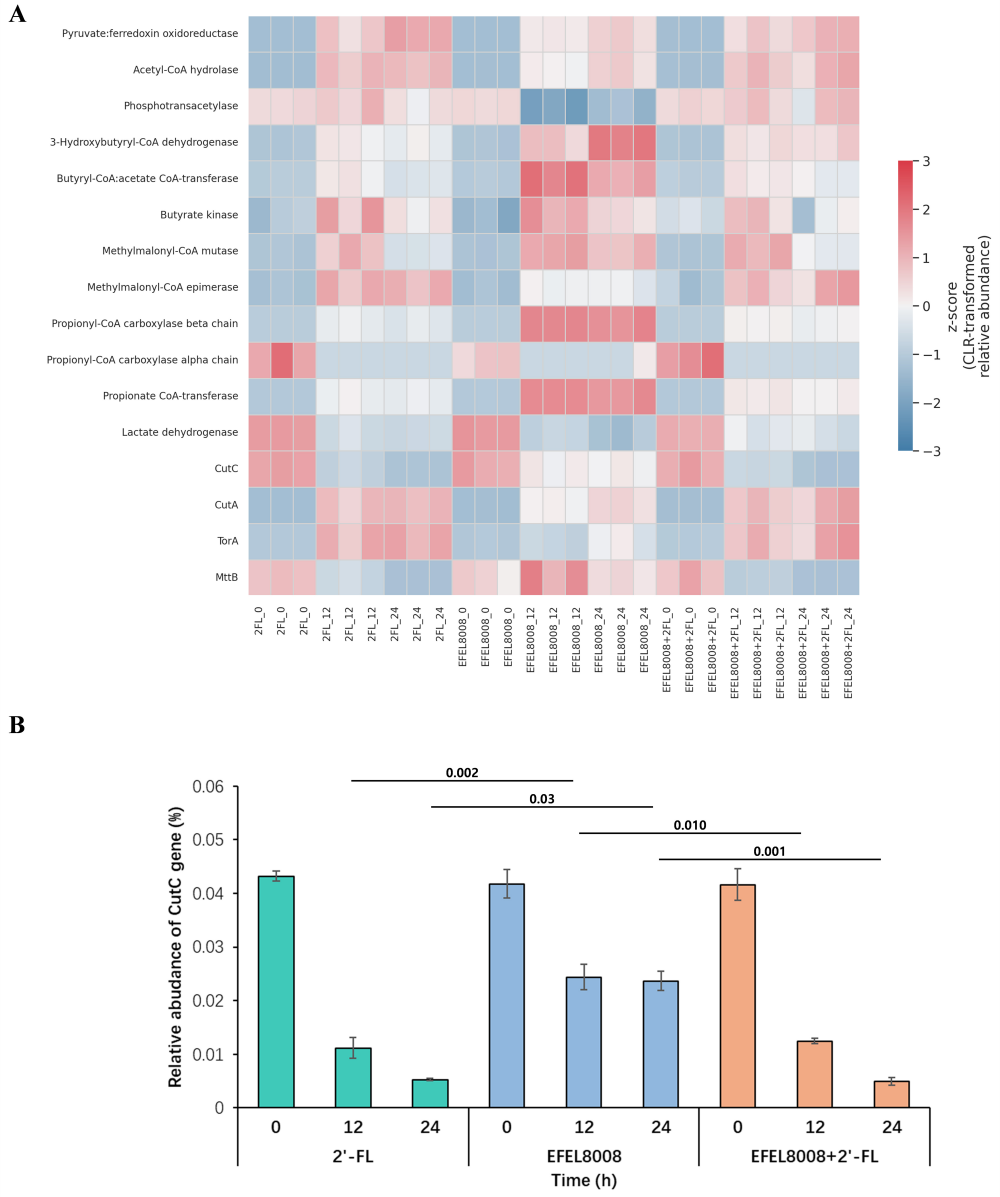
Functional prediction of microbial genes associated with SCFA metabolism and TMA production using PICRUSt2. (A) Heatmap showing the CLR-transformed Z-scores of KEGG orthologs related to microbial metabolic pathways during *in vitro* fecal fermentation. Genes involved in SCFA metabolism and TMA production are visualized across experimental groups and time points; (B) Relative abundance of the *CutC* gene across fermentation groups at 0, 12, and 24 h. Error bars indicate the standard deviation of three biological replicates (*n* = 3). Statistical significance was evaluated using *t*-test, and *P*-values are indicated above the bars. SCFA: Short-chain fatty acid; TMA: trimethylamine; CLR: centered log-ratio transformation; KEGG: Kyoto Encyclopedia of Genes and Genomes.

To assess potential functional changes in microbial metabolism, PICRUSt2-based gene prediction was performed using 16S rRNA sequencing data. KEGG orthologs related to SCFA metabolism were selected and visualized as a heatmap based on CLR-transformed Z-scores [Figure 7A]. Genes associated with SCFA biosynthesis, such as *butyryl-CoA:acetate CoA-transferase*, *butyrate kinase*, and *propionate CoA-transferase*, showed higher predicted abundance in the EFEL8008 + 2′-FL group at 24 h compared to other groups. To further explore genes linked to TMA production, the relative abundance of *CutC* was quantified across time points. Notably, *CutC* abundance was significantly lower in the EFEL8008 + 2′-FL group than in the control group at 24 h (*P* < 0.05; Figure 7B). While these predictions are limited to computational inference, the results suggest that the synbiotic treatment may support increased fermentative capacity and potentially suppress microbial gene abundance associated with TMA biosynthesis.

## DISCUSSION

While the combination of probiotics and prebiotics is recognized as a promising approach to promote gut health through synbiotic effects, identifying ideal pairings that specifically enhance microbial composition and metabolic activity remains a challenge. This study demonstrates that 2′-FL plays a key role in modulating gut microbiome composition, promoting SCFA production, and reducing TMA levels under *in vitro* fecal fermentation conditions, while co-administration with *B. infantis* EFEL8008 may additionally affect these changes.

Prebiotics are defined as non-digestible food components that selectively stimulate the growth or activity of beneficial gut bacteria, particularly in the large intestine, where they exert health benefits^[16]^. To provide their beneficial effects on gut health, prebiotics must withstand enzymatic hydrolysis and digestion throughout the gastrointestinal tract and reach the colon intact^[37]^. In this study, the gastrointestinal stability of 2′-FL was evaluated using an *in vitro* digestion model. As shown in Table 1, 2′-FL was slightly hydrolyzed by salivary amylase and pancreatic amylase, while a small proportion (11.60%) was further degraded by BBMV during the intestinal phase. These findings are consistent with previous reports, where approximately 5% of HMOs were digested after incubation with BBMV^[29]^. 2′-FL has a structure of Fuc-α-1,2-Gal-β-1,4-Glc. The α(1→2) glycosidic linkage can potentially be cleaved by BBMV enzymes^[38]^. However, only limited degradation occurred, likely due to low substrate specificity and restricted enzyme accessibility. These results indicate that the majority of ingested 2′-FL reaches the large intestine intact. This supports its role as an effective prebiotic.

*In vitro* fecal fermentation offers distinct advantages over human clinical trials. The application of *in vitro* fecal fermentation reduces costs, accelerates experimental outcomes, lowers labor requirements, and minimizes ethical concerns. The use of controlled experimental conditions and high reproducibility enables precise mechanistic investigations^[39,40]^. *In vitro* fecal fermentation models are considered effective tools for assessing the responses of gut microbiota to various substrates. Previous studies have demonstrated that prebiotic interventions, such as FOS, inulin, and seaweed polysaccharides, not only promote the growth of beneficial bacteria including *Lactobacillus* and *Bifidobacterium*, but also influence other aspects of the gut microbiota such as metabolite production and community composition, with patterns closely matching those observed in human clinical studies^[30,41,42]^. Based on these advantages, an *in vitro* fecal fermentation model was applied in this study to evaluate the synbiotic effects of 2′-FL and *B. infantis* EFEL8008. During *in vitro* fecal fermentation using 2′-FL as the sole carbon source, Actinobacteria and Bacteroidetes showed the greatest increases at the phylum level, while *Bifidobacterium* and *Bacteroides* were significantly enriched at the genus level [Figure 2]. In contrast, supplementation with *B. infantis* EFEL8008 alone, without a fermentable substrate, did not result in a significant increase in *Bifidobacterium* spp. These findings reflect the limitations of administering probiotics alone. Probiotic strains administered to humans are often transiently detected in feces only during and shortly after administration, failing to establish long-term colonization in the gut^[43,44]^. Colonization resistance, driven by competition for resources and inhibitory interactions, further hampers the ability of single probiotics to modulate the indigenous microbiota^[45,46]^. In contrast, supplementation with prebiotics such as 2′-FL provides targeted nutrients that may enhance the proliferation of administered probiotics within the fecal microbiota.

Prebiotic supplementation supports the colonization of beneficial strains and promotes restructuring of the gut microbiota toward a more favorable composition^[47]^. In this study, 2′-FL alone significantly increased the relative abundance of *Bifidobacterium* compared to the control during *in vitro* fecal fermentation. This observation aligns with previous reports demonstrating the bifidogenic effect of 2′-FL in *in vitro* models using fecal samples from individuals with irritable bowel syndrome (IBS)^[48]^. The increase in *Bifidobacterium* induced by 2′-FL supplementation may further support cross-feeding interactions within the gut microbial community. *Bifidobacterium* species, including *B. infantis*, possess multiple α-fucosidases capable of releasing L-fucose from 2′-FL^[49,50]^. The liberated L-fucose provides a carbon source for other commensals such as *Bacteroides*, which may explain the concurrent increase in *Bacteroides* abundance observed in this study [Figure 2B]. Collectively, these results highlight that synbiotic interventions combining 2′-FL and *B. infantis* not only enhance the engraftment of beneficial bacteria but also promote beneficial shifts in the gut microbial ecosystem.

To evaluate the synbiotic effects of EFEL8008 and 2′-FL, it was necessary to detect and monitor target microbes throughout the complex microbiome fermentation process to ensure reproducibility. For this, culture-independent methods such as RT-qPCR have been developed to enumerate individual species from complex DNA samples by quantifying the number of copies of target genes^[51]^. Accurate classification of *B. longum* at the subspecies level using 16S rRNA gene sequences remains challenging in NGS-based metagenomic sequencing due to high genetic similarity among subspecies^[52,53]^. In this study, a primer specific to *B. infantis* was developed based on the sialidase gene (EC:3.2.1.18) [Supplementary Figure 1]. As a result, RT-qPCR analysis using the strain-specific primer demonstrated that EFEL8008 + 2′-FL resulted in a greater increase in *B. infantis* compared to EFEL8008 or 2′-FL alone [Figure 5]. Although *B. infantis* has been extensively studied for its beneficial effects during infancy, evidence regarding its adaptability in adult hosts remains limited. *B. infantis* is a predominant member of the gut microbiota in breastfed infants but is rarely detected in adults^[11,54]^. In the present *in vitro* fecal fermentation study, *B. infantis* alone did not proliferate effectively in adult fecal conditions. However, when administered together with 2′-FL, *B. infantis* exhibited increased abundance within the adult gut microbiota during *in vitro* fermentation. These results suggest that synbiotic strategies combining *B. infantis* with specific prebiotics such as 2′-FL may enhance the colonization efficiency of *B. infantis* in adult populations. Further human intervention studies are warranted to substantiate these findings and to explore the long-term health implications of *B. infantis*-based synbiotic approaches.

During *in vitro* fecal fermentation, acetate, lactate, propionate, and butyrate were identified as the major organic acids produced [Figure 6A-D]. In the human gut, lactate is typically utilized by cross-feeding bacteria such as *Eubacterium hallii* and *Anaerostipes* spp*.*, which are often underrepresented *in vitro* models^[55]^. Despite this limitation, the measured concentrations of SCFAs still provide meaningful insights into the fermentation capacity and metabolic shifts induced by 2′-FL and EFEL8008. This observation is consistent with previous findings from continuous colon simulator models, where acetate and lactate were the predominant SCFAs produced during synbiotic fermentation^[11,56]^. Propionate plays an important role in weight control and glucose homeostasis by inhibiting hepatic lipogenesis and, along with acetate, stimulates free fatty acid receptor 2 (FFAR2), thereby suppressing the secretion of ghrelin, an appetite-stimulating hormone^[57]^. Additionally, acetate and lactate can serve as substrates for cross-feeding by other beneficial gut microbes, particularly butyrate-producing Firmicutes species such as *Anaerostipes*, *Eubacterium*, and *Faecalibacterium*^[11]^. Consistent with this mechanism, our fermentation results also demonstrated a significant increase in butyrate levels following the synbiotic treatment with 2′-FL and *B. infantis* EFEL8008 [Figure 6D]. The resulting increase in butyrate production is particularly important, as butyrate serves as a primary energy source for colonocytes and provides multiple health benefits, including strengthening the gut barrier, exerting anti-inflammatory effects, and inhibiting the growth of pathogenic bacteria^[58]^. In addition, TMA was less converted than probiotics or prebiotics after fermentation from betaine [Figure 6E and F]. Carnitine and choline can be converted by gut microbiota into TMA via betaine compound using carnitine dehydrogenase or choline dehydrogenase^[25]^. The reduced levels of TMA observed under the combined treatment may be attributed to the suppression of *Escherichia* and *Clostridium*, genera that are frequently associated with microbial genes responsible for TMA production, such as *cutC* and *cntA*^[59,60]^. This compositional shift likely contributed to the decreased microbial conversion of betaine to TMA. In support of this, functional gene prediction using PICRUSt2 revealed a significantly lower relative abundance of *cutC* in the EFEL8008 + 2′-FL group compared to the control at 24 h. Although this result is based on computational inference and does not confirm gene expression or enzymatic activity, it provides additional support for the potential suppression of TMA-related microbial pathways under synbiotic treatment. Betaine can be obtained from various foods, mainly regulates lipid metabolism, glucose homeostasis, and is known to have a good effect on immune regulation^[61]^. Numerous studies, including the present investigation, have reported that betaine is metabolized into TMA by gut-residing microbes. Once generated, TMA is absorbed through the intestinal epithelium and transported to the liver, where it is further oxidized to TMAO via hepatic flavin-containing monooxygenases. Subsequently, TMAO is systemically distributed and may accumulate in peripheral tissues or be excreted through the kidneys. This metabolite has been implicated in the development of cardiovascular diseases, including atherosclerosis and myocardial infarction^[26]^. Therefore, the results of this study suggest that the synbiotic strains used may have the potential to impact CVD-related factors, highlighting the importance of further research to explore these effects in more detail. These findings suggest that the synbiotic combination of EFEL8008 and 2′-FL may influence adult gut microbial metabolism under defined conditions. However, whether intentional modulation of the gut microbiota in healthy individuals is truly beneficial remains uncertain^[62]^. Nevertheless, the observed metabolic responsiveness indicates that infant-derived probiotics, such as EFEL8008, may functionally engage with adult microbiota without disrupting ecosystem balance. This highlights the potential of EFEL8008 and 2′-FL as a promising synbiotic strategy for microbiota-targeted applications.

This study was conducted using an *in vitro* fecal fermentation model, which does not fully capture the complexity of the human gastrointestinal environment. In particular, the batch fermentation system lacks continuous removal of metabolic byproducts and the activity of key cross-feeding microbes, often leading to artificial accumulation of intermediate metabolites such as lactate and a consequent reduction in pH. This study also did not include an unsupplemented control group, which may have limited the interpretation of treatment-specific effects relative to baseline microbial activity. The exclusion of an unsupplemented control group was due to the limited capacity of the batch fermentation system, which restricted the number of experimental conditions that could be implemented in parallel using the same fecal donor. Based on this constraint, treatment groups were selected according to their relevance to the study objectives, with a focus on interactions between the probiotic and prebiotic components. In future studies, an unsupplemented control group will be included to improve interpretation against baseline microbial activity. In addition, the current *in vitro* platform will be extended to *ex vivo* and *in vivo* models to further validate synbiotic effects under more physiologically relevant conditions. Despite these limitations, the results provide mechanistic insights into how dietary components may influence microbial metabolic outputs. Notably, the addition of 2′-FL supported the growth of *B. infantis* EFEL8008 and other beneficial taxa within adult fecal microbiota under fermentation conditions. While *B. infantis* alone showed limited proliferation, its abundance increased substantially in the presence of 2′-FL, suggesting that this specific carbohydrate source may facilitate the expansion of infant-derived strains even in adult-associated microbial environments. Furthermore, co-treatment with 2′-FL and EFEL8008 led to increased levels of acetate, propionate, and butyrate, alongside a reduction in the microbial production of TMA. These functional changes indicate that the selected combination may promote a more favorable metabolic profile in the gut environment under defined conditions. Collectively, these findings support the potential of targeted nutritional strategies to modulate the functional capacity of the adult gut microbiota.

## DECLARATIONS

### Availability of data and materials

The data supporting this work are provided in the text. Additional raw data will be made available by the corresponding author upon reasonable request.

### Copyright

© The Author(s) 2025.
