# Supplementary material for: Synbiotic modulation of adult gut microbiome by 2′-fucosyllactose and Bifidobacterium longum subsp. infantis EFEL8008
Source: Microbiome Res Rep. 2025 Jul 22;4(3):26. doi: 10.20517/mrr.2025.35 (PMC12540058; doi:10.20517/mrr.2025.35)
Supplement: Supplementary file 1 [file mrr-4-3-26-SupplementaryMaterials.pdf]

Supplementary Materials

Synbiotic modulation of adult gut microbiome by 2′-fucosyllactose and *Bifidobacterium longum* subsp. *infantis* EFEL8008

Dong Hyeon Lee[#], Hyunbin Seong[#], Seul-Ah Kim, Nam Soo Han

Director of Brain Korea 21 Center for Smart GreenBio Convergence and Sustainable Regional Development, Division of Animal, Horticultural, Food Sciences, Chungbuk National University, Cheongju 28644, Republic of Korea.

[#]Authors contributed equally.

Correspondence to: Prof. Nam Soo Han, Director of Brain Korea 21 Center for Smart GreenBio Convergence and Sustainable Regional Development, Division of Animal, Horticultural, Food Sciences, Chungbuk National University, S-20 606, 1 Chungdae-ro, Cheongju 28644, Republic of Korea. E-mail: namsoo@cbnu.ac.kr

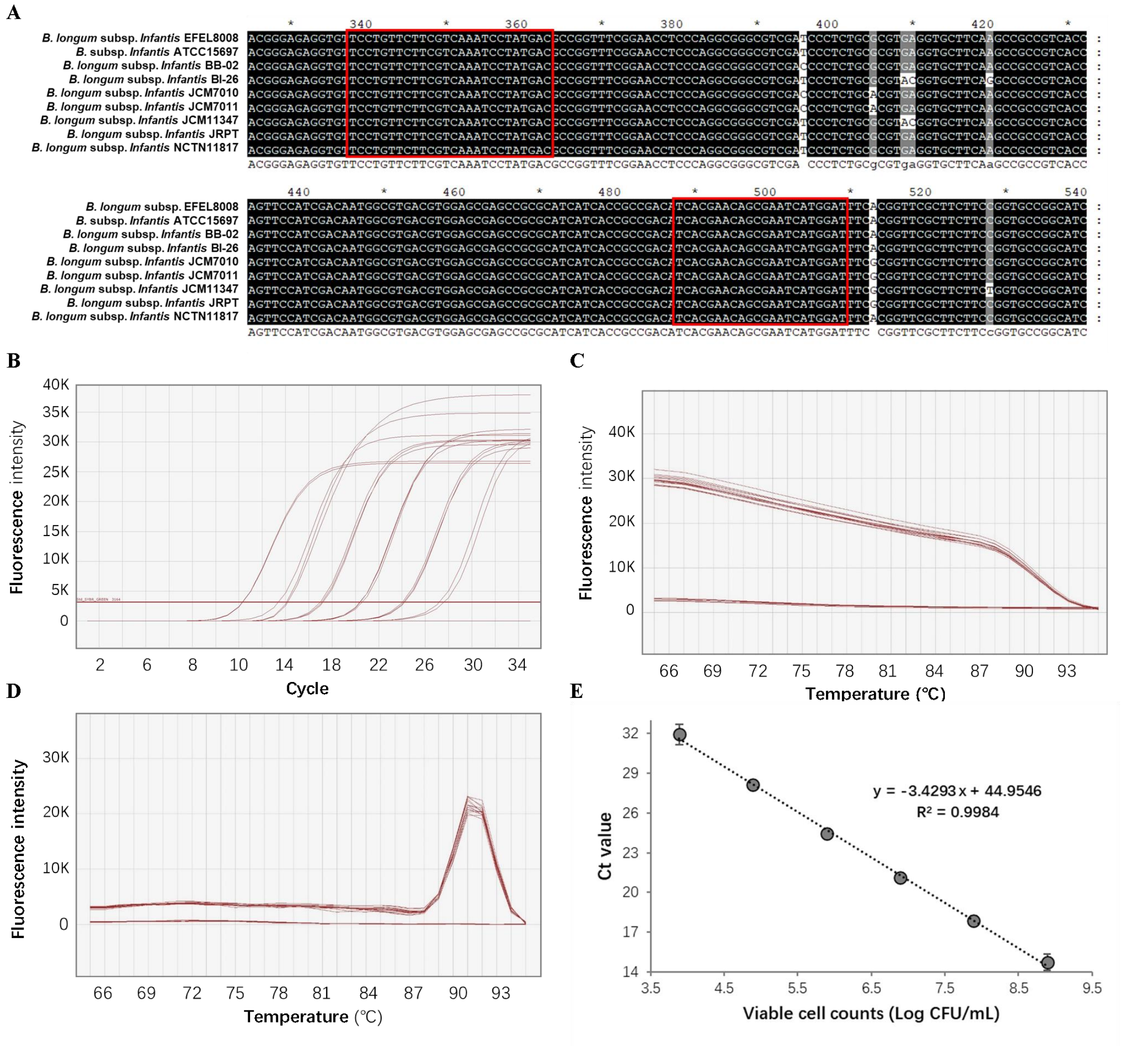

Supplementary Figure 1. Development and validation of real-time qPCR method for *Bifidobacterium longum* subsp. *infantis* detection. (A) Homologous region alignment of the sialidase gene across nine *B. infantis* strains showing sequence conservation and primer binding sites; (B) Ct curve illustrating the amplification kinetics; (C) Melting curve analysis confirming primer specificity; (D) Melting peak graph showing a single distinct peak for the target amplicon; (E) Standard curve demonstrating linearity of detection across five orders of magnitude (R^2 = 0.9984).
